# Is periapical surgery follow-up with only two-dimensional radiographs reliable? A retrospective cohort type sensitivity study

**DOI:** 10.4317/medoral.24447

**Published:** 2021-10-27

**Authors:** Amparo Ramis-Alario, Beatriz Tarazona-Álvarez, Miguel Peñarrocha-Diago, David Soto-Peñaloza, María Peñarrocha-Diago, David Peñarrocha-Oltra

**Affiliations:** 1 DDS, MS. Master in Oral Surgery and Implantology. Stomatology Department, Valencia University Medical and Dental School, Valencia, Spain; 2 DDS, PhD. Postdoctoral Associate Professor. Stomatology Department, Valencia University Medical and Dental School, Valencia, Spain; 3 MD, PhD. Chairman of Oral Surgery and Director of the Master in Oral Surgery and Implantology. Stomatology Department, Valencia University Medical and Dental School, Valencia, Spain; 4 DDS, MS, PhD. Master in Oral Surgery and Implantology. Stomatology Department, Valencia University Medical and Dental School, Valencia, Spain; 5 MD, DDS, MS, PhD. Full Professor of Oral Surgery. Stomatology Department, Valencia University Medical and Dental School, Valencia, Spain

## Abstract

**Background:**

Two-dimensional (2D) radiographic techniques are commonly used for assessing lesion prognosis after endodontic surgery. The present retrospective cohort study analyzes the sensitivity and ability of different radiographic techniques in obtaining area and volume measurements of periapical lesions.

**Material and Methods:**

Preoperative and follow-up (6-48 months) periapical and panoramic radiographs (index test) and cone-beam computed tomography (CBCT) images (reference standard) were selected from an endodontic microsurgery database. Sensitivity was analyzed independently by two examiners. The areas of the 2D radiographic images and CBCT volumes were studied using Itk-Snap software and Romexis viewer.

**Results:**

The sample comprised 105 patients and 105 teeth, with a mean follow-up of 21 months (range 6-48). Preoperatively, CBCT detected all the periapical areas, periapical radiography detected 67, and panoramic radiography detected 60. Postoperatively, of the 52 cases in which CBCT detected remains of the periapical area, periapical radiography detected 22, and panoramic radiography detected 17. The measurements of the areas obtained by the 2D methods, and the volumes obtained by CBCT, had to be transformed into linear measures for comparison purposes. The measurements were found to be significantly different in both the preoperative and the follow-up images.

**Conclusions:**

Periapical radiography showed greater sensitivity than panoramic radiography, both preoperatively and at follow-up. The lesions measured with CBCT were larger, with significant differences than as evidenced by the periapical and panoramic radiographs.

** Key words:**Periapical radiography, panoramic radiography, CBCT, sensitivity, treatment outcome, size of periapical radiolucency, area, volume.

## Introduction

The radiographic changes in persistent apical periodontitis are a characteristic sign of this disorder (1), and it is very important to know the extent of the periapical bone defects before endodontic microsurgery to facilitate treatment and improve the prognosis of the tooth (2). Such changes range from the widening of the periodontal ligament to a well-defined periapical area (3).

At present, periapical radiography is the most common technique used in application to persistent apical periodontitis, both for the initial diagnosis and for follow-up (4,5). Panoramic radiography is not as frequently used as periapical radiography, though it does offer a comprehensive and rapid two-dimensional (2D) view of the jaws, making it easy to use in daily clinical practice (6).

The main drawbacks of two-dimensional diagnostic methods in their reliability rely in its limited information about size, extension and location of the periapical lesion (7), because of compression of three-dimensional structures, geometric distortion and anatomic noise obscuring diagnostic clarity of the region of interest (8) . Moreover, in the vestibular plane of periapical radiographs, the information provided is limited due to bone superimposition that makes it difficult to observe periapical radiolucent areas (4). In addition, the size of the periapical radiolucency may be affected by the orientation of the film and tube head (9).

Some authors associate the size of the periapical lesion to the prognosis of endodontic surgery, reporting better results with smaller areas (10,11), while other investigators have found no relationship between previous size and the prognosis of the tooth (12,13). In any case, prior to endodontic microsurgery, it is important to precisely establish the extent of the lesion in order to optimize the surgical procedure (2). During the follow-up, it is also important to assess the size of the periapical area in order to adequately assess the outcome of endodontic microsurgery, especially when the tooth needs to be used for prosthetic purposes.

Cone-beam computed tomography (CBCT) has proven its superiority over two-dimensional radiographs (avoiding anatomical noise, compression of three-dimensional anatomy and geometric distortion, and allowing better assessment of the root canal anatomy) (14,15). Besides, CBCT has been recommended in cases where apical surgery is being considered (16). No studies to date have investigated the sensitivity of the mentioned two-dimensional radiographic techniques (periapical and panoramic) versus CBCT both before surgery and in the course of follow-up. Likewise, no comparisons have been made of the periapical areas obtained with these two-dimensional techniques versus the volumes obtained with CBCT before and after surgical treatment.

The present study was carried out to analyze the sensitivity of two-dimensional radiographic techniques and their reliability in evaluating the size of periapical bone defects, both preoperatively and during the follow-up period.

## Material and Methods

- Study design and setting

The present retrospective cohort type sensitivity study is reported according to the STARD statement (Standards for Reporting of Diagnostic Accuracy Studies) (17).

- Patient selection

A consecutive cohort of 298 patients underwent endodontic microsurgery between January 2015 and January 2018 at the Stomatology Department of the University of Valencia (Valencia, Spain). The cohort was evaluated and screened for study eligibility by the first author (A.R.A.) before study entry.

Inclusion criteria: Patients with a single tooth subjected to primary endodontic microsurgery, involving the use of three radiological diagnostic methods (CBCT, periapical radiography and panoramic radiography) affording images of sufficient quality to allow linear measurements of the periapical lesions, and who reported for follow-up examinations after 6-48 months postsurgery.

Exclusion criteria: The adoption of regenerative procedures (bone grafts, membranes).

- Endodontic microsurgery

Modern microsurgical techniques were performed in all the patients (18). High magnification was used with a rigid endoscope (Karl Storz-Endoskope, Tuttlingen, Germany), with ultrasonic preparation (Piezon® Master 400, EMS®, Electro Medical Systems S.A., Switzerland) and root-end fillings with mineral trioxide aggregate (MTA) (ProRoot MTA White, Dentsply Tulsa Dental, Tulsa, OK, USA). All surgeries were carried out by the same oral surgeon (M.P.D.).

- Imaging techniques

Index test: Periapical and panoramic radiographs:The periapical radiography images were obtained with a Gendex expert D unit (Gendex Dental Systems, Hatfield, USA). A phosphor-plate system was used (Vista Scan®, Dürr Dental AG, Bietigheim-Bissingen, Germany). Images were taken according to the parallel technique using a film holder (Super-Bite®, Kerr, Bioggio, Switzerland). The exposure parameters were: 65 kV, 7 mA and 0.080.12 sec. The distance between the sensor and X-ray tube was 30 cm. Images were processed and stored by the processor DBSWIN (Dürr Dental, Germany).

The panoramic radiographs were obtained with a Planmeca ProMax 3D Classic CBCT unit (Planmeca, Helsinki, Finland), and the Planmeca Romexis® application was used for processing the images (version 4.5.2; Planmeca). The exposure parameters were: 68 kV, 10 mA and 19 sec.

Reference standard: CBCT: The CBCT images were obtained with a Planmeca ProMax 3D Classic CBCT unit (Planmeca, Helsinki, Finland), and the Planmeca Romexis® Viewer application was used for processing the images. A limited field of view (FOV) (40 x 40 mm) with 12 sec duration at 90 kV, 8 mA and voxel size 0.15 mm was used for CBCT imaging.

- Test sensitivity

For both the two-dimensional radiographic techniques and CBCT, a periapical lesion was defined as a periapical radiolucency in contact with the apical part of the root that exceeded at least twice the width of the periodontal ligament space. Also, in CBCT images, the lesion had to be visible in at least two image planes (16). The sensitivity of the two-dimensional techniques (index test) compared with CBCT (reference standard) was assessed according to the imaging stage involved (preoperative and follow-up). All preoperative and follow-up images were evaluated independently by the first author (A.R.A) and a blinded assessor (D.P.O.). Disagreements were resolved by discussion with a third advisor (D.S.P.). The two-dimensional radiographic images were evaluated first, followed by the CBCT images at least two weeks later.

Examiners were calibrated, and the appraisals were conducted under standardized conditions.

- Lesion area and volume calculation

The radiographic images were evaluated jointly by the same two examiners (A.R.A. and D.P.O.). During the measurements, the examiners discussed and reached a consensus regarding the limits of the areas. Previously, to ensure calibration of measures and inter-examiner reliability, 10 images per radiographic technique from patients not involved in the present study and presenting periapical areas were evaluated twice, with a one-week interval.

**Figure 1:**
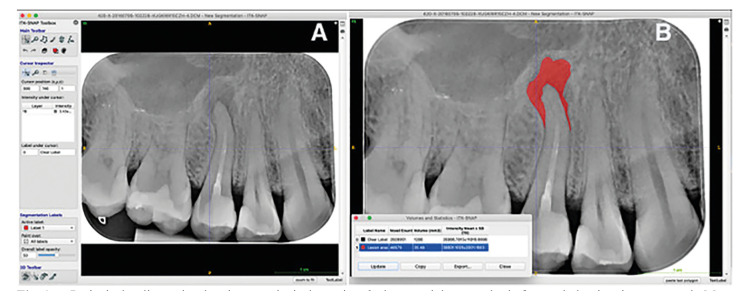
a. Periapical radiography showing a periapical area in a 2nd upper right premolar before endodontic microsurgery. b. Measurement performed manually in the program Itk-Snap (20.49 mm2).

**Figure 2:**
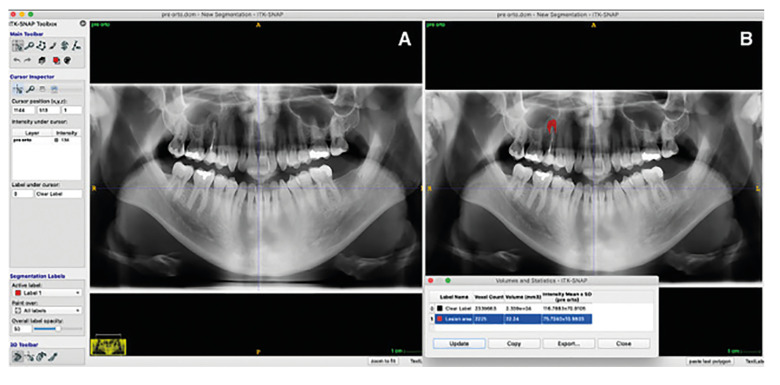
a. Panoramic radiography showing the periapical area in the same 2nd premolar. b. Measurement performed manually in the program Itk-Snap (22.24 mm2).

Periapical and panoramic radiographs assessment: The area measurements were done using the Itk-Snap program (free access software, http://www.itksnap.org). Two investigators (A.R.A and D.P.O) used this program to measure the images preoperatively and follow-up in square millimeters. The examiners made manual tracing. The freehand selection was used to trace out the lesion contour (after calibrating the software's scale according to the size of the active sensor area). Then, the area value was measured and recorded as previously reported (19). If a multiple root tooth had more than one periapical lesion, the individual defects were calculated and then added together to obtain a total defect area (20) (Fig. 1, Fig. 2).

**Figure 3:**
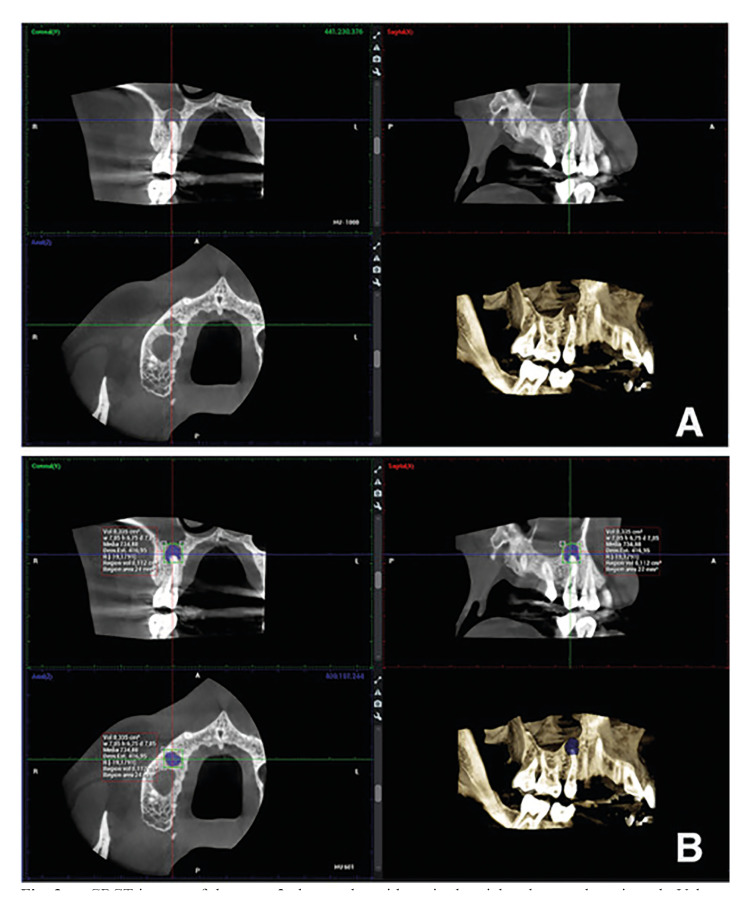
a. CBCT images of the same 2nd premolar with sagittal, axial and coronal sections. b. Volume measurements performed in the Planmeca Software (112 mm3).

Tomographic imaging assessment: For the volume measurements in the CBCT images, the dedicated Planmeca Romexis® application (version 4.5.2; Planmeca) was used. This software allowed volume calculation of the periapical bone defects through manual tracing of the border of the lesion across the different slices (axial, coronal and sagittal), which contained the bone defects and, finally, the program calculated the total volume in cubic millimeters (Fig. 3).

Standardization assessment: All images were evaluated on a 21.5-inch monitor (iMac; Apple, Cupertino, CA, USA) with a screen resolution of 4096 x 2304 pixels and located in a quiet room with subdued lighting.

- Statistical analysis

The SPSS version 15.0 (Chicago, IL, USA) statistical package was used, with the level of significance being set at 0.05. The McNemar test for 2x2 repeated measures was applied to analyze the sensitivity of the two-dimensional radiographic techniques versus CBCT. Differences in linear measurements were estimated using a t-test for paired comparisons.

In the sensitivity analysis, the agreement between both examiners was calculated based on the Cohen kappa statistic and interpreted according to the Landis and Koch scale (21). The sensitivity of both two-dimensional methods was compared using CBCT as the reference standard in identifying the lesions, in line with previous studies (5,22,23). Sensitivity was also analyzed against the different teeth, considering incisors, canines, premolars and molars, and tooth arch (maxilla and mandible).

Concerning linear measurement capacity, intra- and inter-examiner repeatability yielded a mean rating (K=2) indicating absolute agreement, using a two-way mixed-effects model intraclass correlation coefficient (ICC), and interpreted according to Fleiss scale (24).

Indirect comparison was made to compare the two-dimensional radiographic methods with CBCT: the square roots of the two-dimensional radiography measurements and the cubic roots of the CBCT measurements were calculated (20).

## Results

Between January 2015 and January 2018, a total of 298 patients were referred for endodontic surgery performed by the same oral surgeon (M.P.D.). After applying the inclusion and exclusion criteria, the final sample consisted of 105 patients (105 teeth): 33 males and 72 females, with a mean age of 48.8 ± 16 years, and with a mean follow-up of 21 months (range 6-48).

The kappa scores for the preoperative and follow-up periapical images were k=0.933 and k=0.924, respectively, indicating almost perfect agreement between the two examiners. In the preoperative and follow-up panoramic images, the kappa values were k=0.895 and k=0.829, respectively. In contrast, in the CBCT images, for healing evaluation, the kappa value in the follow-up images was k=0.981.

Periapical radiography versus CBCT:Of the 105 preoperative images in which CBCT detected a periapical area, periapical radiography identified a periapical area in 67 cases (64%, with a false-negative rate of 36%, resulting in a sensitivity of 64%) (Table 1). The 38 cases not detected by periapical radiography had a volume of between 4-419 mm3.

At follow-up, where CBCT detected a periapical area in 52 cases, periapical radiography detected a periapical area in 22 cases (42%, with a false-negative rate of 58%, resulting in a sensitivity of 42%) (Table 1).

No differences were found when sensitivity was compared across the different groups of teeth (incisors, canines, premolars and molars) in the periapical images, either at preoperative or follow-up, and the differences between the maxilla and mandible were not significant.

Panoramic radiography versus CBCT: Preoperatively, CBCT detected 105 teeth with periapical areas, while panoramic radiography detected 60 periapical areas (57%, with a false-negative rate of 43%, resulting in a sensitivity of 57%) (Table 1). The 45 cases not identified by panoramic radiography had a volume between 6-484 mm3.

During the follow-up period, of the 52 periapical areas identified by CBCT, panoramic radiography identified 17 cases (33%, with a false-negative rate of 67%, resulting in a sensitivity of 33%) (Table 1).

As in the periapical images, no significant differences were found on comparing the sensitivity between different groups of teeth. However, significant differences were observed between the maxilla and mandible in the preoperative images, with greater sensitivity for the mandible. In the follow-up panoramic radiographs, no differences were observed between the maxilla and mandible.

Lesion area and volume calculation: The intraclass correlation coefficient (ICC) was 0.897, 0.832 and 0.857 for periapical radiography, panoramic radiography and CBCT, respectively, indicating a very good ICC.

The mean area measured on the preoperative periapical radiographs was 18.8 mm2 (n=105, standard deviation [SD] ± 29.2). The mean size in the preoperative panoramic radiographs was 17.6 mm2 (n=105, SD±34.6). The same cases measured by CBCT yielded a mean preoperative volume of 141.1 mm3 (n=105, SD±244.7). In the follow-up images, periapical radiography yielded 2.9 mm2 (n=105, SD±6.7), panoramic radiography 2.3 mm2 (n=105, SD±6.2) and CBCT 21.8 mm3 (n=105, SD±45.7).

When the square roots of the periapical radiographs were compared with the cubic roots of the images obtained by CBCT, the results showed CBCT to yield measurements significantly larger than those of periapical radiography, both preoperatively (n=105, mean=1.11, SD±1.97, *p*<0.001) and during the follow-up period (n=105, mean=0.80, SD±1.25, *p*<0.001). When the square roots of the panoramic radiographic images were compared with the cubic roots of the CBCT images, the results yielded statistically significant differences between the two techniques both preoperatively (n=105, mean=1.45, SD±2.47, *p*<0.001) and in the follow-up images (n=105, mean=0.94, SD±1.58, *p*<0.001) (Table 2). No significant differences were seen on comparing periapical radiography with panoramic radiography.

**Table 1:**
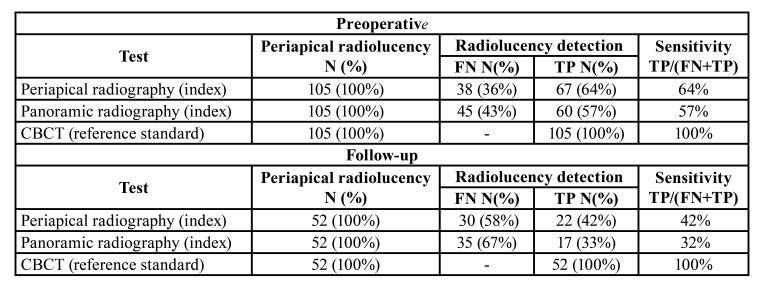
Rating of radiolucency in periapical radiography, panoramic radiography and CBCT, absolute values and percentages, both preoperatively and control period.

**Table 2:**
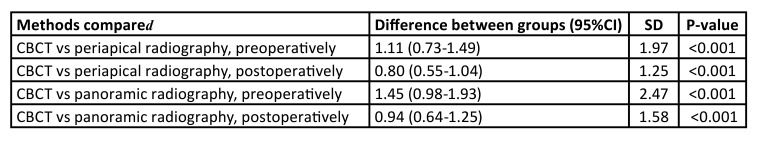
Results of the t-test for dependent samples according to the evaluation method used, with the corresponding 95% confidence interval, standard deviation and *p-value*.

## Discussion

The present retrospective sensitivity study evaluated endodontic microsurgery prognosis in a cohort after 6-48 months of follow-up. Comparing the periapical and panoramic radiographs versus the CBCT images yielded a sensitivity of under 65% in all cases for both two-dimensional techniques, both preoperatively and follow-up. Likewise, significant differences were found in comparing the measurements of the periapical areas carried out by the two-dimensional methods and CBCT.

Endodontic surgery is very predicTable, though follow-up is crucial. Periapical radiography is normally used for follow-up. Nevertheless, many authors have shown that two-dimensional radiography does not have the same sensitivity as CBCT (5,16). In this study, sensitivity and the capacity to perform linear measurements of the lesions with the two-dimensional techniques were evaluated preoperatively and during the follow-up and were compared with CBCT.

Both two-dimensional radiographic techniques (periapical and panoramic) were analyzed since they are widely available and employed in dental clinics and have been used in many other studies (5,22,25,26).

In the preoperative images, periapical radiography detected 64% of the periapical areas detected by CBCT, while panoramic radiography detected 57%. Another study, including periapical and panoramic radiography (25) found periapical radiography to identify 64% of the periapical areas detected by CBCT, while panoramic radiography detected 55.5% of the lesions detected by CBCT. These data are similar to those obtained in our study and likewise evidence the superiority of periapical radiography over panoramic radiography. Estrela *et al*. (5) also compared periapical and panoramic radiography, obtaining lower sensitivity performances than in the present study (54.5% for periapical radiography and 27.8% in the case of panoramic radiography).

Fewer studies to date have compared sensitivity between periapical radiography and CBCT in follow-up images (27-29), and no studies have contrasted sensitivity between panoramic radiography and CBCT after surgery.

In the follow-up images, CBCT and periapical radiography showed 70.5% agreement on the diagnosis of the presence or absence of a periapical area, while 26.7% of the follow-up periapical areas were only detected by CBCT. These percentages are quite similar to those obtained by Christiansen *et al*. (27), who recorded agreement between both methods in 67% of the cases. In 28% of the cases were diagnosed by CBCT but not for periapical radiography in the follow-up images. It should be noted that their study was limited to radiographic images obtained one year postoperatively. Results similar to our own were published by Kruse *et al*. (28), in a sample of 74 teeth, in which disagreement between periapical radiography and CBCT was seen in 27% of the cases. However, their mean duration of the follow-up period was longer than in our study.

Von Arx *et al*. (29) conducted a study in humans comparing images one year after periapical surgery, observing differences between CBCT and periapical radiography in 40.5% of the cases. This Figure is higher than that obtained in the present study, was 29.5% disagreement was observed between CBCT and periapical radiography. The differences between the two studies could be explained by the fact that the former authors did not dichotomize their results into two groups but classified them into three categories: no radiolucency present, scar type radiolucency, and lesion type radiolucency.

Regarding panoramic radiography, the latter technique coincided with the CBCT findings in 65% of the cases in our series - this Figure being a little lower than the 70% agreement rate obtained between CBCT and periapical radiography.

The measurements obtained with two-dimensional radiography were compared against CBCT, based on the calculation of the square roots (two-dimensional radiography) and cubic roots of the measurements (CBCT), following the previous descriptions article (20). The results were statistically significant for comparing periapical radiography and CBCT, with larger values being obtained with CBCT. Gouveia *et al*. (30) likewise compared the measurements obtained in a sample of 11 patients, comparing the area of the periapical radiograph and a central mesiodistal section of the CBCT scan in mm2. These authors compared the postoperative images after 48 hours and four and 8 months, with no comparison of the preoperative images. No statistically significant differences were observed between the methods in any of the periods analyzed, though the method used to obtain the measurements differed from that employed in our study.

We identified only one previous study about panoramic radiography, which measured the periapical area and compared it with CBCT (26). The measurements were made differently, with the area being calculated as if it were a rectangle - no significant differences being found.

It would be interesting to follow-up on apical surgery with CBCT because it has been widely demonstrated that the sensitivity of two-dimensional radiographic methods is limited. To do so, however, the radiation doses must be well defined and adjusted in each case to avoid excessive radiation exposure.

It should be mentioned that the final sample included in our study might not be representative of a normal population subjected to apical surgery, since in many cases, follow-up CBCT is only completed because the patient has some kind of symptom. Consequently, the follow-up CBCT scan could suggest a poorer prognosis than if it had been made in the absence of symptoms.

Cone-beam computed tomography detected all the periapical lesions, while periapical and panoramic radiography showed a poor detection rate, leading to an increased misdiagnosis incidence. Both two-dimensional methods yielded measurements significantly different from those obtained by CBCT.
